# Supplementary figures and images for: Variability of tissue mechanical response in Sus Domesticus porcine models from in vivo to ex vivo conditions
Source: PLoS One. 2023 May 10;18(5):e0268608. doi: 10.1371/journal.pone.0268608 (PMC10171650; doi:10.1371/journal.pone.0268608)

**S1 Fig. Measured Temperatures of Liver and Peritoneum Pre- and Post-Testing.**

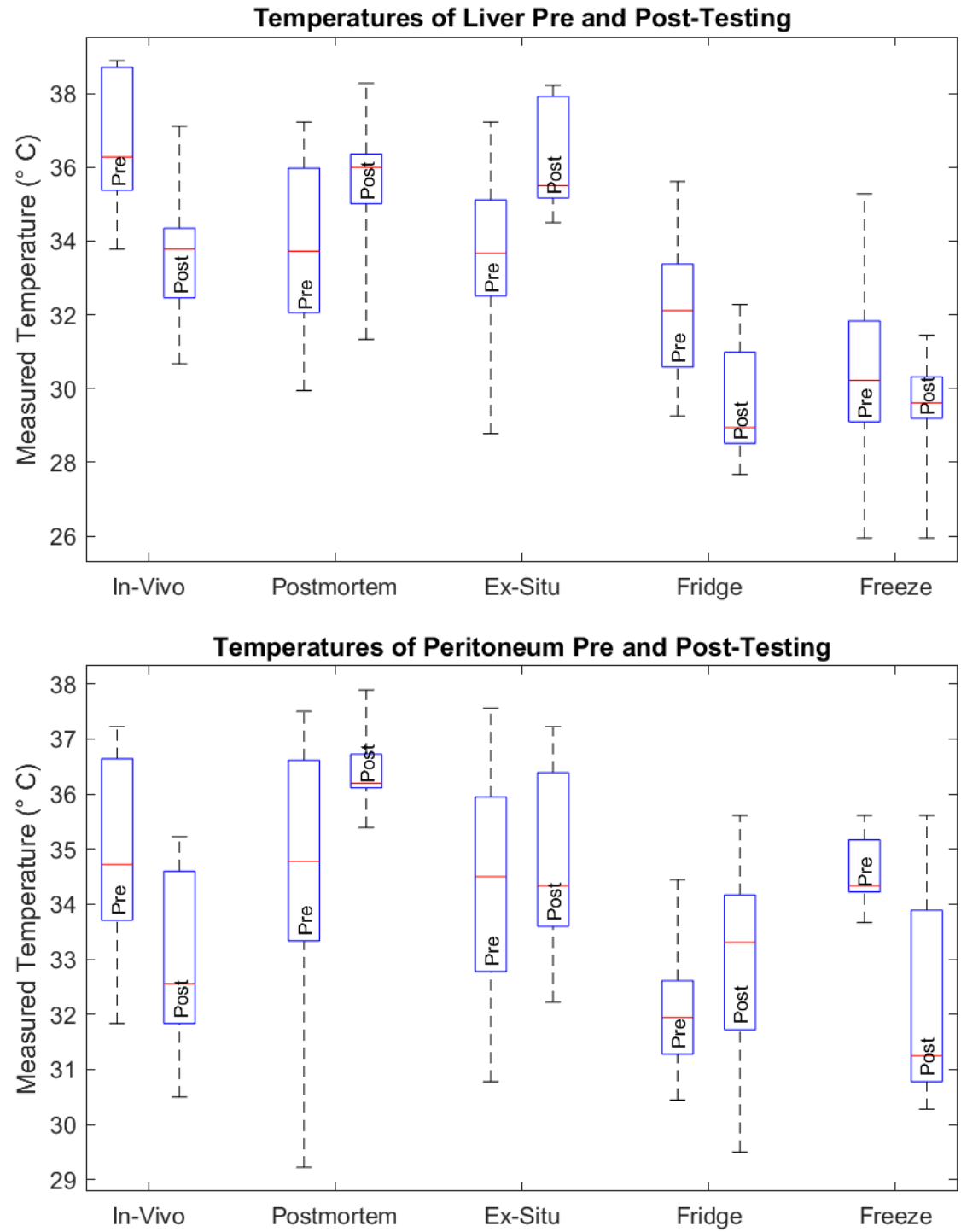

Supplement: S1 Fig — (PDF) [file pone.0268608.s001.pdf]

**S4 Fig. Grasper Test on Calibration Puck.**

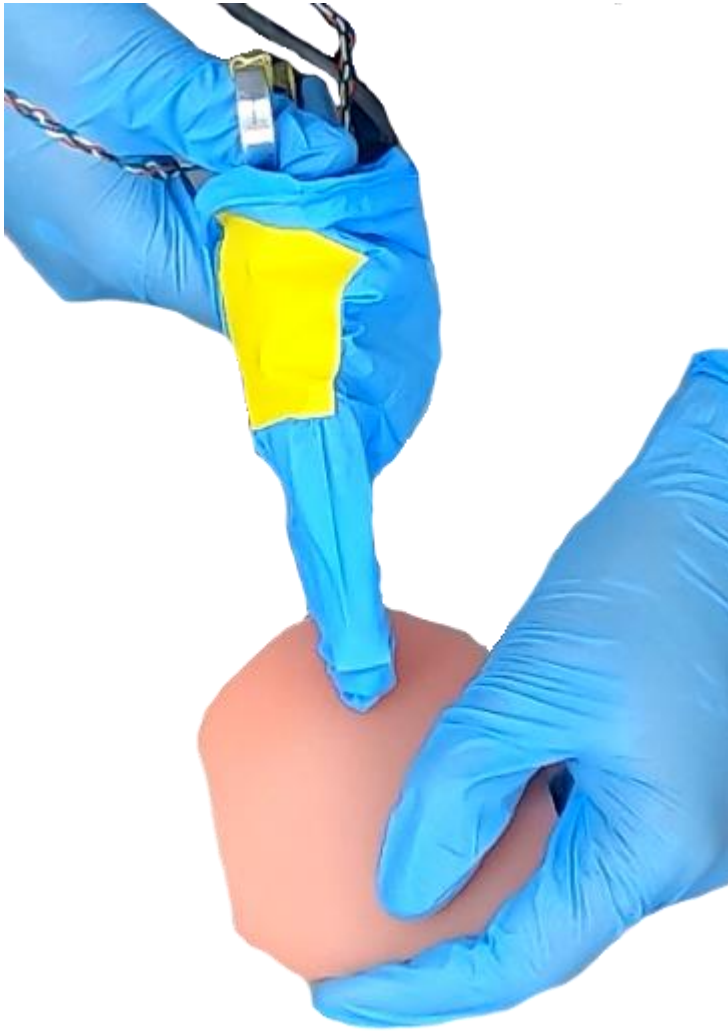

Supplement: S3 Fig — (PDF) [file pone.0268608.s003.pdf]
