# Supplementary material for: Variability of tissue mechanical response in Sus Domesticus porcine models from in vivo to ex vivo conditions
Source: PLoS One. 2023 May 10;18(5):e0268608. doi: 10.1371/journal.pone.0268608 (PMC10171650; doi:10.1371/journal.pone.0268608)
Supplement: S2 Fig — (PDF) [file pone.0268608.s002.pdf]

S3 Supporting Information. Grasper Device Details.

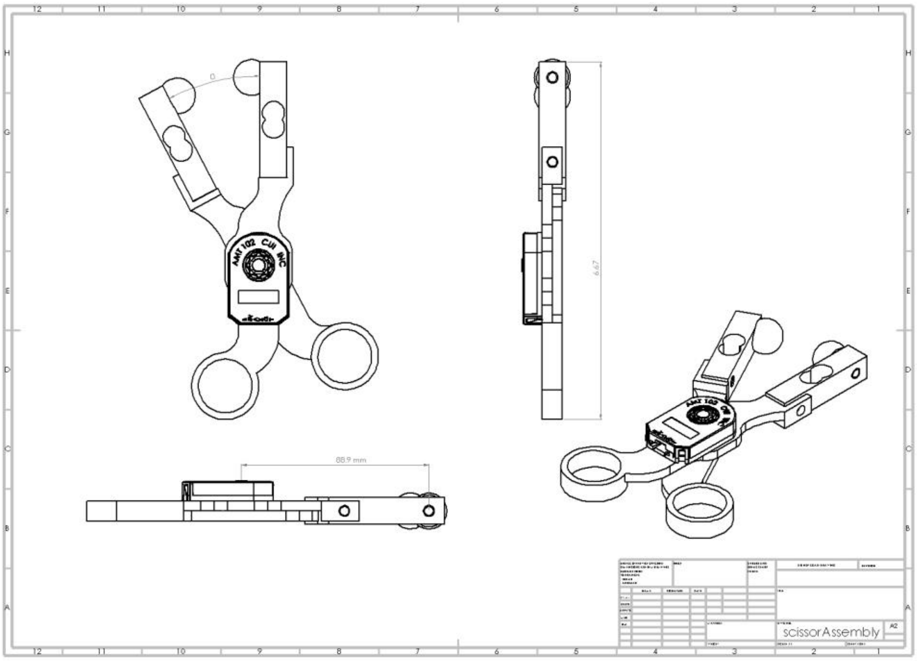

The scissor-like grasper device used in this study collects two raw input values consisting of voltages from the 5kg load cells at the grasper jaws and encoder tick position. The voltages from the load cells are converted to compressive force in Newtons applied to the grasped tissue via a calibration process. At the beginning and end of each testing session, a standard set of weights was applied to each grasper jaw and the input voltage was recorded. The values were then plotted and a line of best fit was generated in which the slope correlated to the voltage to force conversion for all results analyses for that session. This calibration process was repeated for every testing session.

In addition to converting the recorded voltages from the load cells to forces, it is also necessary to convert the resulting encoder tick position to a usable measurement value. For this study, the encoder tick position was converted to angular position of

the encoder jaws, which could then be converted to the overall tissue thickness between the grasper jaws. For the encoder used on this grasper device, a full 360° range of motion was 2048 encoder ticks, thus the angular position of the grasper jaw could be determined via 5.688 encoder ticks per degree of rotation. With the angular position of the grasper known and the overall radius of the grasper jaw from tissue point of contact to encoder position known, the angular position of the encoder was correlated to the distance between grasper jaws, which is equivalent to tissue thickness during the grasping process.

This overall grasper device design was chosen for its capability to monitor both applied compressive loads to the tissue and tissue thickness simultaneously during the grasping process. It was also chosen for its flexibility of being implemented across various testing conditions.

The load cells were connected to IAA100 Analog Strain Gauge Signal Conditioners (Futek, one per load cell) that amplified the voltage signals. The signal conditioners are also adjusted so the voltage reading was zero when the grasper was held in the "neutral" position.

The output from the signal conditioners were fed into the National Instruments Data Acquisition card (NI DAQ, PCIe 6320 model), which was configured to read the differential voltage from each signal conditioner. The signal lines from the encoder were also connected to the DAQ, which was configured to read and interpret the digital signals from the encoder and convert them into encoder ticks.
