# Supplementary material for: Variability of tissue mechanical response in Sus Domesticus porcine models from in vivo to ex vivo conditions
Source: PLoS One. 2023 May 10;18(5):e0268608. doi: 10.1371/journal.pone.0268608 (PMC10171650; doi:10.1371/journal.pone.0268608)
Supplement: S4 Fig — (PDF) [file pone.0268608.s004.pdf]

S5 Supporting Information. Curve Fitting Methodology.

**Initial Guess**	Alpha	Beta
1	-2	5
2	-1	2.5
3	-0.25	4
4	-0.03	6.5
5	-0.5	6.5
6	-20	1

All initial guesses were used for all curves in order to avoid falling into local minima.

Curves were fit to the following equation:

$$F(t) = \alpha * \exp\,(\beta * \epsilon(t) - 1)$$

Where $F(t)$ is the force observed during the grasp, after five-sample window gaussian smoothing in order to minimize noise and facilitate better curve fitting. *Epsilon* $\epsilon(t)$ is the strain observed during the grasp, and α and β are the curve fit variables.

Consequently, we derive a "stiffness" value using the following equation:

$$Stiffness = \frac{dF(t)}{d\epsilon(t)} = \alpha * \beta * \epsilon(t) * \exp\big(\beta * \epsilon(t)\big)$$

Thus, we obtain a stiffness value at a particular force level by finding the point in time where the force value is closest to the desired force value (5N or 6.5N), then using the corresponding strain at that point in time, and the curve fit α and β values in the equation above.

A "pseudo" R-squared value was calculated for each fit curve using the sum-squared residual (SS_{resid}) and sum-squared total (SS_{total}) as follows:

$$Quality = 1 - \frac{SS_{resid}}{SS_{total}}$$

Where SS_{total} is calculated as:

$$SS_{total} = \sum_{t=1}^{t_{final}} (F(t) - F_{mean})^2$$

Where F_{mean} is the mean force value throughout the grasp.

A minimum quality threshold of 0.95 was used to exclude any grasps where curve fitting was not deemed to be sufficiently accurate.
