# Supplementary material for: Variability of tissue mechanical response in Sus Domesticus porcine models from in vivo to ex vivo conditions
Source: PLoS One. 2023 May 10;18(5):e0268608. doi: 10.1371/journal.pone.0268608 (PMC10171650; doi:10.1371/journal.pone.0268608)
Supplement: S5 Fig — (PDF) [file pone.0268608.s005.pdf]

S6 Supporting Information. Load Cell Calibration.

The load cells were each calibrated at least before and after a testing day using a series of known weights from 10 grams to 500 grams, including a data point with no weights applied. In order to validate the voltage measurements, we applied a linear curve fit through the data points, mapping the measured voltage to the known applied force.

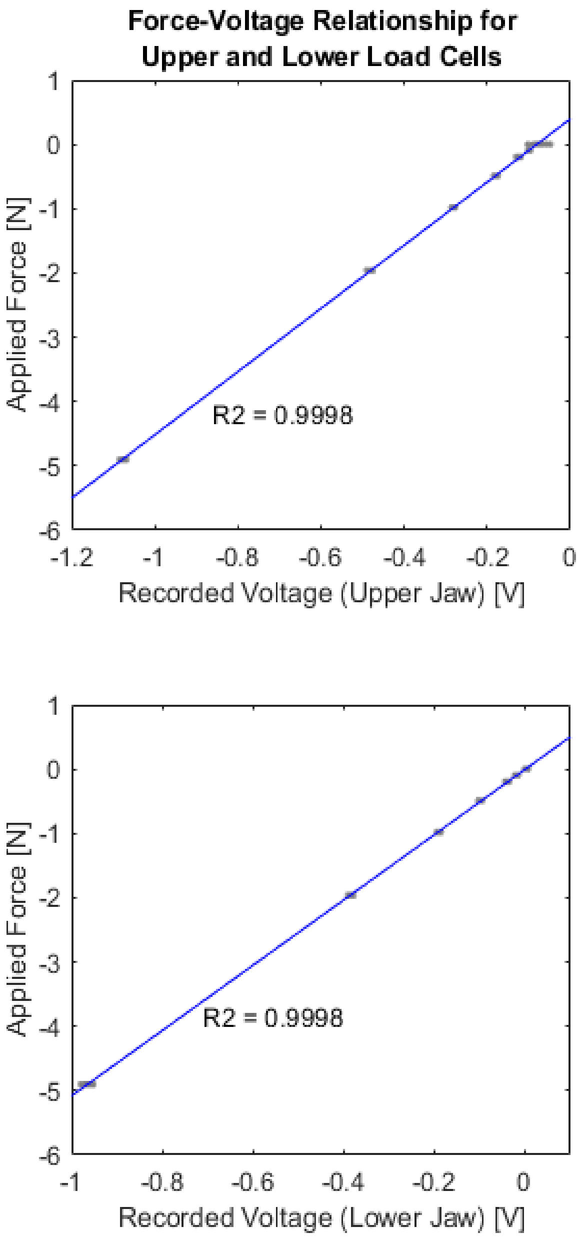
Force-Voltage Relationship for
Upper and Lower Load Cells
Applied Force [N]
R2 = 0.9998
Recorded Voltage (Upper Jaw) [V]
1
0
-1
-2
-3
-4
-5
-6
-1.2
-1
-0.8
-0.6
-0.4
-0.2
0
Applied Force [N]
R2 = 0.9998
Recorded Voltage (Lower Jaw) [V]
-1
-0.8
-0.6
-0.4
-0.2
0

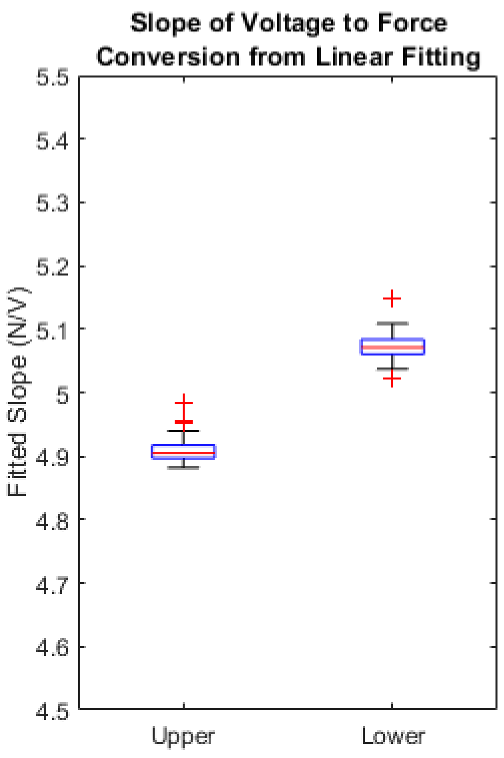

Voltage to Force linear fits have R-squared values above 0.99 for all fits, and the slope of the conversion (in Newtons per Volt) is consistent across calibration sessions. Variation can be explained by noise and sometimes non-perpendicular application of force to the load cell.
