# Supplementary material for: Variability of tissue mechanical response in Sus Domesticus porcine models from in vivo to ex vivo conditions
Source: PLoS One. 2023 May 10;18(5):e0268608. doi: 10.1371/journal.pone.0268608 (PMC10171650; doi:10.1371/journal.pone.0268608)
Supplement: S6 Fig — (PDF) [file pone.0268608.s006.pdf]

S7 Supporting Information. Methodology Validation Using Calibration Puck.

Grasping was performed on the calibration puck before the first tissue, in between each tissue, and after the final tissue tested. Force-displacement curves were generated and curve-fitting applied using the same methods as for the tested tissues.

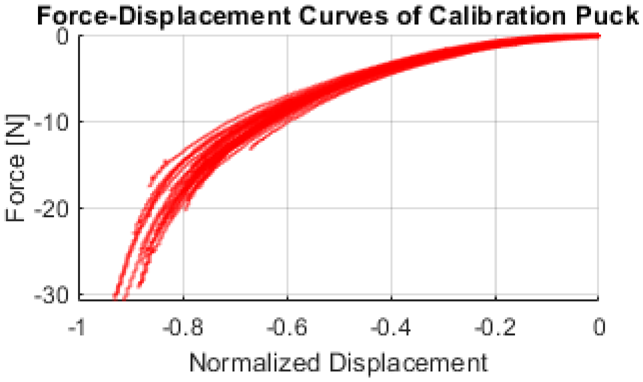

Plot of the “raw” Force vs. Displacement for the Calibration puck. The apparent “stair-stepping” of the curves is caused by quantization noise from the encoder.

Calibration Puck data shows some variation, despite being the exact same material with same measurement methods. Variation can be partially explained by grasping near the edges (apparent material stiffness can change due to edge effects), and grasping the puck at an angle.

Next, we quantify the stiffness of the puck at various force values. The results indicate no statistically significant differences between any of the testing sessions.

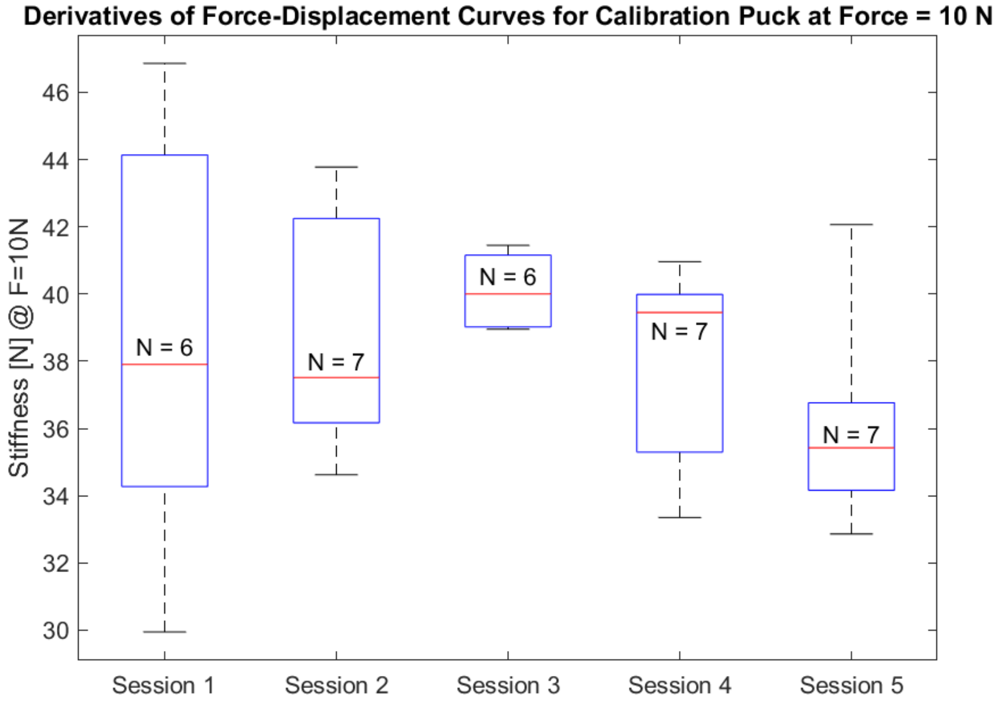
Derivatives of Force-Displacement Curves for Calibration Puck at Force = 10 N
Stiffness [N] @ F=10N
30
32
34
36
38
40
42
44
46
N = 6
N = 7
N = 6
N = 7
N = 7
Session 1
Session 2
Session 3
Session 4
Session 5

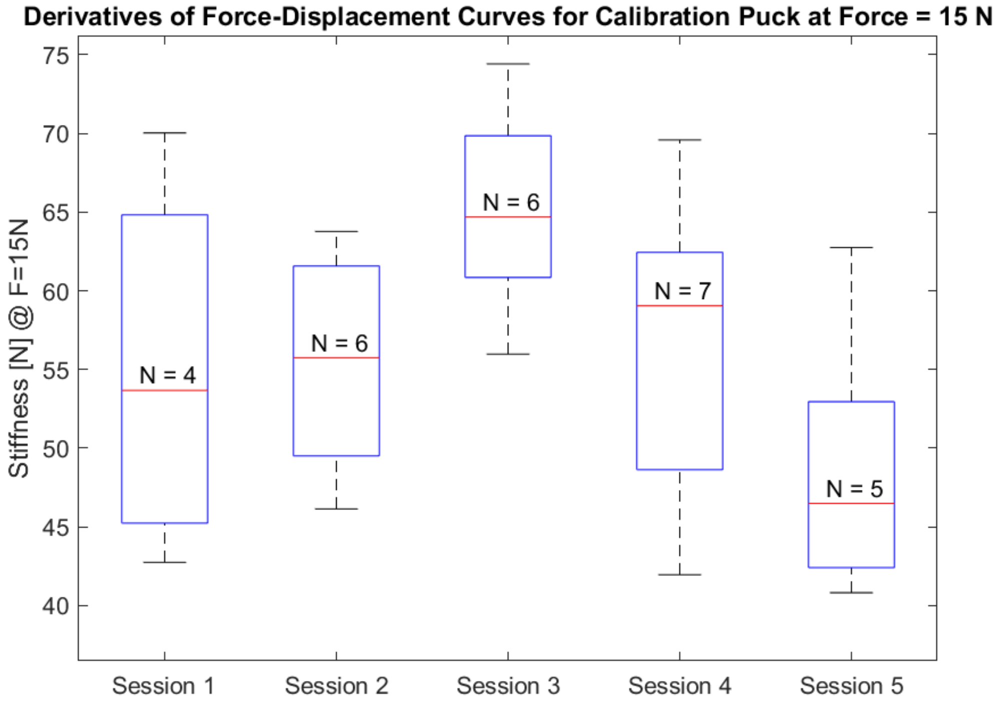
Derivatives of Force-Displacement Curves for Calibration Puck at Force = 15 N
Stiffness [N] @ F=15N
40
45
50
55
60
65
70
75
N = 4
N = 6
N = 6
N = 7
N = 5
Session 1
Session 2
Session 3
Session 4
Session 5

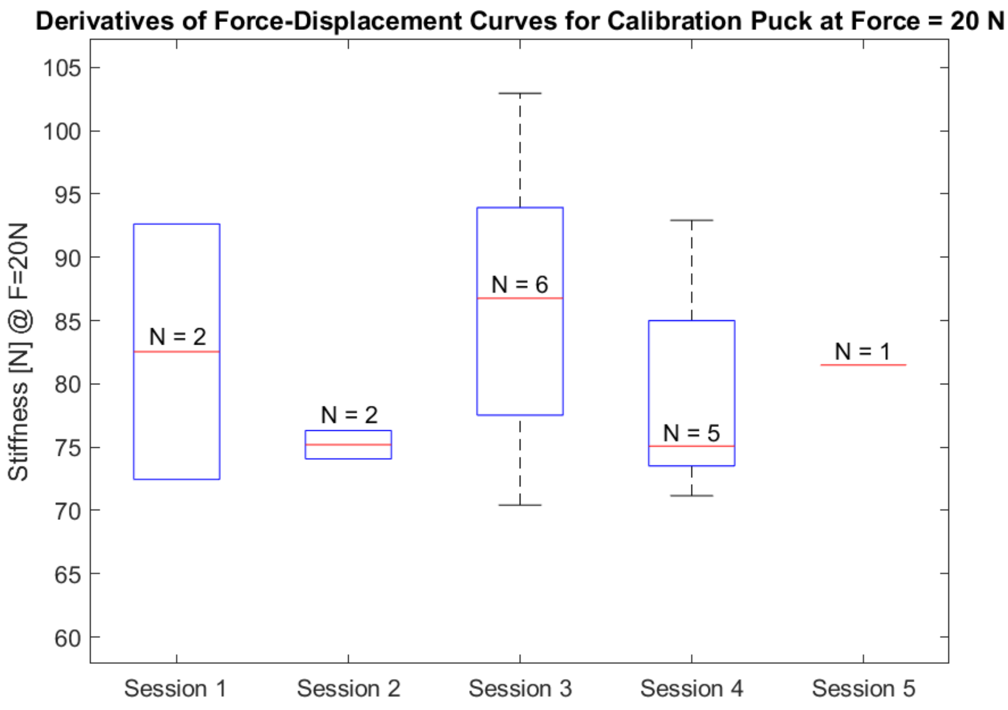

Whiskers encompass all data points; outlier marking is disabled.
