# Supplementary material for: Variability of tissue mechanical response in Sus Domesticus porcine models from in vivo to ex vivo conditions
Source: PLoS One. 2023 May 10;18(5):e0268608. doi: 10.1371/journal.pone.0268608 (PMC10171650; doi:10.1371/journal.pone.0268608)
Supplement: S1 File — (PDF) [file pone.0268608.s007.pdf]

S2 Link. Scissors Console Source Code.

https://github.com/labmrd/Scissors_Console
