# Supplementary material for: Variability of tissue mechanical response in Sus Domesticus porcine models from in vivo to ex vivo conditions
Source: PLoS One. 2023 May 10;18(5):e0268608. doi: 10.1371/journal.pone.0268608 (PMC10171650; doi:10.1371/journal.pone.0268608)
Supplement: Attachment — Submitted filename: Response to Reviewers.pdf [file pone.0268608.s008.pdf]

Dear Dr. Viegas,

Thank you for giving us the opportunity to submit a revised draft of the manuscript “Variability of Tissue Mechanical Response in Sus Domesticus Porcine Models from in vivo to ex vivo Conditions“ for publication in PLOS ONE. Our team appreciates the time and effort that you and the reviewers have dedicated to providing feedback on our manuscript. We have incorporated most of the suggestions made by the reviewers and believe that your recommendations have provided valuable improvements to our manuscript. The changes our team has made are highlighted within the tracked changes version of the manuscript. Provided below is a point-by-point summary of each comment made by the reviewers along with the research teams response as well as how that comment was addressed in the revised manuscript.It should be noted that all line and page numbers that reference the manuscript are for the revised manuscript file with tracked changes.

All of the authors thank you for your time and feedback.

Editor Comments to the Authors:

Introduction:

Editor Comment: Pay attention to Latin terms such as in vivo which must be written in italics.

Author Response: We appreciate your comment for exposing this oversight, and have adjusted the text accordingly. All instances of latin terms have been italicized based on your recommendation.

Editor comment: Post mortem in Latin should be written in italics. The authors use the Latin expression and the expressions postmortem or post-mortem that in my opinion should not be used.

Author Response: The team appreciates the feedback, and have adjusted our formatting of the expression in the text accordingly. All instance of the verbiage “postmortem” or “port-mortem” have been changed to “”post mortem” and have been italicized.

Editor Comment: Statistical p should also be written in italics.

Author Response: Thank you for pointing this out! We have adjusted our text accordingly and have italicized statistical p whenever it appears in the manuscript.

Editor Comment: Why do you write post-freeze in italics?

Author Response: The team initially used “post-freeze” in italics to highlight it as a tissue state, however we have adjusted our usage of italics, as suggested by your feedback as it should not be in italics for this usage.

Editor Comment: Table 2 is not previously announced in the text.

Author Response: As suggested by the editor, we have introduced a reference to Table 2 in the text. The team has added the following passage to the manuscript at the end of the “Research Objective and Contributions of this Paper” section, beginning in line 150:

“Tables 2 and 3 compare and summarize the cited prior works, as well as the presented study.”

Material and Methods:
Editor Comment: Although Table 3 is a repository of the sample of animals used, the sample must be briefly described in advance.

Author Response: Thank you for this suggestion. We have added the following text prior to the table (formerly table 3, now table 4) in order to more accurately describe the sample population:

“The population for this study consisted of five Yorkshire-X porcine animals. The population size of five was chosen to ensure that inter-animal variability could be represented in our data set, while minimizing the number of animals needed. Weights of each animal ranged from 71.2kg to 83.0kg while the animals ranged between 119 days to 154 days (4 to 5 months) in age. An overview of the study population is shown in Table 4 below.”

Editor Comment: The in vivo testing procedure is not described.

Author Response: Thank you for pointing out the exclusion of this information in the original manuscript submission. The research team has added the in vivo testing procedure, as well as a more detailed data collection methodology for all tissues in each tissue state in a new section, titled “Usage”, which begins in line 298.

Editor Comment: I am of the opinion that the description of the functioning of the test device should be made after the description of the sample and prior to the description of the studies.

Author Response: While the team appreciates your feedback, the team believes that the current organization of the “Materials and Methods” section presents the methods in a narrowing scope that would allow the reader to more easily understand the details at various depths. Additionally, with the addition of the “Usage” subsection, we believe the text flows better into the “Results” section.

Results:
Editor Comment: Line 295 – we are talking about experimental animals, not patients

Author Response: Thank you for pointing out this error in verbiage. The team has adjusted all appropriate instances where the term patient was used when referring to experimental animals.

Discussion:
Editor Comment: It is supposed in this section to compare our work with previous works

Author Response: Based on this comment, as well as a comment from Reviewer 2, the discussion has been expanded upon and adjusted to include additional discussion, particularly regarding comparing this work with previous work mentioned in the introduction. Again, the research team would like to thank you for all of your feedback.

Reviewers' Comments to the Authors:

Reviewer 1

Reviewer Comment: A number of issues exist with this manuscript which I will briefly point out.

Author Response: Thank you for your comments! We attempted to address them to the best of our ability.

Reviewer Comment: 1. There is no discussion

Author Response: The team agrees that our discussion section was quite limited and have greatly expanded it thanks to your feedback. We have added a passage that better integrates our results with the prior works mentioned in the introduction.

Reviewer Comment: 2. Introduction is a collection of paragraphs each describing one study

Author Response: Thank you for pointing this out. The team chose to present our introduction in such a manner in order to chronologically introduce the substantial background of this topic, and so we could highlight the specific contributions of our work.

Reviewer Comment: 3. no statistical analysis is described within the graphs

Author Response: The team agrees with the reviewer, and have accordingly added a short paragraph to the beginning of the results section to elaborate how the statistical analysis was performed. We also reorganized the text in order to more clearly outline the key findings and methods.

Reviewer Comment: 4. table summarizing previous studies (table 2) would benefit from more detail describing the outcome of those studies.

Author Response: The research team agrees with this recommendation and have added an additional table to the manuscript (table 3) which discusses major outcomes and findings of each study.

Reviewer Comment: 5. Table 3 would benefit to know the age of the animals (not haveing to calculate it one's self).

Author Response: Thank you for this recommendation. The research team has added a column to the study population overview table, formerly table 3 now table 4 in the revised manuscript, which includes the age of each animal in days. Similarly, the research team has also adjusted the dates of birth and session start dates to have consistent formatting. All dates are now in the form of YYYY/MM/DD.

Reviewer 2

Reviewer Comment: Dear author: Congratulations for your effort in the preparation of this article. Reading the document I have some doubts I hope that you resolve me.

Author Response: Thank you! We appreciate your feedback and have attempted to incorporate your comments into our manuscript.

Reviewer Comment: Lines 94-100. Rewrite the paragraph. You repeat sitiffness and viscosity twice in a short time. In my opinion not neccesary.

Author Response: While the team acknowledges that the terms stiffness and viscosity are repeated in this passage, the team has decided that both instances should be included in the final manuscript. A modification to the exact verbiage was made such that the phrase "stiffer and more viscous" is not repeated. The updated text when discussing study findings now reads "viscosity and stiffness of the tissue increases" instead of "the tissue becomes stiffer and more viscous". The team believes this modification keeps the original clarity of the passage while avoiding repetition of specific verbiage.

Reviewer Comment: Line 104. Were the differences significant or not? I have not clear if it is mistoken.

Author Response: The team has added clarification to the text signifying that findings from the study by Brouwer et al. found a statistically significant discrepancy among fit parameters. The verbiage "statistically significant" has been added for clarity.

Reviewer Comment: Line 177. Please, chnage the commercial name of the drug for the correct (Tiletamine and zolazepam).

Author Response: Thank you for pointing this incorrect drug name usage out. Telazol has been changed to Tiletamine/Zolazepam in the manuscript. Additionally, the team has gone through the entire manuscript to verify no commercial drug names are used in the text and the correct names are used.

Reviewer Comment: In addition, I have some doubts about the protocol:

Author Response: We appreciate your concern about the protocol. We have clarified our "Materials and Methods" section according to your feedback.

Reviewer Comment: 1. I miss an anagesia protocol, specially in thoracic surgeries. The anesthesia is not enough and this may produce changes in the tissues caused by the shock.

Author Response: Thank you for pointing this out, as this information should be included directly in the manuscript. The team has added a passage in the “Anesthesia” section that reads:

“Throughout testing, Visible Heart Lab (VHL) professionals with anesthesia training were present and monitored for signs of shock or inadvertent emergence from anesthesia.”

We hope that this included passage adds the necessary justification and clarification for the analgesia protocol. The protocol was designed, implemented, and overseen in coordination with anesthesia professionals from the Visible Heart Lab.

Reviewer Comment: 2. You should explain what other procedures were done in these animals, because may have influence in your results

Author Response: The team agrees with your point in that the additional procedures done in these animals should be acknowledged in more detail. Unfortunately, the team does not have this information accessible currently; however, a short passage has been added to the text in the “Euthanasia” section that reads as follows:

“Any additional procedures, as part of other studies, were planned accordingly by the VHL to minimize risk of cross study and procedure effects.”

Similar to the analgesia protocol, VHL staff assisted in coordinating across projects and resource requests to ensure, to the best of their ability, that there would be minimal risk of cross study and procedure effects. This staff was present throughout the testing process and monitored the status of the animal to look for any adverse effects from other procedures.

Reviewer Comment: 3. How was calculated the sample size. If not, you should indicate it.

Author Response: The team agrees that this information should be included in the final manuscript and has been added. Thank you for pointing out this lack of detail. The following text has been added in the “Porcine Models and Tissue Handling” section which reads as follows:

“The population size of five was chosen to ensure that inter-animal variability could be represented in our data set, while minimizing the number of animals needed.”

Ultimately the number of animals for use in this study was determined by two primary factors which include: having enough animals to ensure statistically significant claims both within animals and across animals, and minimizing the number of animals used as part of this study. Minimizing the number of animals was a primary concern both from an ethics standpoint as well as to minimize resource requests of VHL.

Reviewer Comment: 4. Why did you administered omega-3 fatty acids in some subjects and not in all. Did you check if this could have influence in the results.

Author Response: Thank you for mentioning this potential concern. We have modified text in the “Anesthesia” section to now read as follows:

“Lipovenous or other combinations of omega-3 fatty acids were administered to all animals as necessary to promote viability *in vitro* in all subjects as part of standard protocol in the VHL”

The research team hopes that this modification, underlined for clarity, provides further explanation on the methodology for administering omega-3 fatty acids. All animals received omega-3 fatty acids to promote viability; however, the amount administered varied by animal as deemed necessary by the VHL staff present monitoring the animal. After further discussions with VHL staff, this is a relatively standard protocol for animals in the VHL to ensure viability across projects/studies in an animal. Ultimately, the use of omega-3 fatty acids were outside the scope of control for the research team as part of this study. After additional research, the team also believes that any effects this could have on the tissue and resulting stiffness’ are minimal and unlikely to be significant.
