# Supplementary material for: Variability of tissue mechanical response in Sus Domesticus porcine models from in vivo to ex vivo conditions
Source: PLoS One. 2023 May 10;18(5):e0268608. doi: 10.1371/journal.pone.0268608 (PMC10171650; doi:10.1371/journal.pone.0268608)
Supplement: Attachment — Submitted filename: Response To Reviewers.pdf [file pone.0268608.s009.pdf]

Dear Dr. Viegas,

Thank you for giving us the opportunity to submit a revised draft of the manuscript “Variability of Tissue Mechanical Response in Sus Domesticus Porcine Models from in vivo to ex vivo Conditions“ for publication in PLOS ONE. Our team appreciates the time and effort that you and the reviewers have dedicated to providing feedback on our manuscript. We have incorporated most of the suggestions made by the reviewers and believe that your recommendations have provided valuable improvements to our manuscript. The changes our team has made are highlighted within the tracked changes version of the manuscript. Provided below is a point-by-point summary of each comment made by the reviewers along with the research teams response as well as how that comment was addressed in the revised manuscript.It should be noted that all line and page numbers that reference the manuscript are for the revised manuscript file with tracked changes.

All of the authors thank you for your time and feedback.

Reviewers’ Comments to the Authors:

Reviewer 3

The scientific paper "Variability of Tissue Mechanical Response in Sus Domesticus Porcine Models from in vivo to ex vivo Conditions" aimed to produce a comprehensive data set by attempting to measure the change in mechanical responses encountered in typical surgical grasping in porcine models between tissue in its native living (in vivo) state and four deceased states.

Thank you for taking the time to review our manuscript and provide your feedback. The research team has made various changes to the manuscript’s discussion based on your feedback which we feel strengthens the overall purpose and validity of the manuscript.

The authors made changes to the manuscript according to the reviewers' suggestions. In my opinion, the discussion is still the weak point of the manuscript, in addition to the scarce number of references used in this section.

Upon further review based on your feedback, as well as other reviewer’s feedback, the research team agrees that the discussion was the weakest section of the manuscript. The research team has reformatted the manuscript to better adhere to PLOS ONE standards and in doing so has restructured and added additional content to the discussion. Instead of presenting the results of the prior work in the introduction, the research team moved it to the discussion section to more directly compare with our results.

Reviewer 4

The present paper is very important for healthcare simulators field as valuable resource for training technical and nontechnical skills.

The research team agrees that the results from this study and manuscript bring attention to an important potential knowledge gap in regard to tissue properties. Thank you for taking the time to review our manuscript and provide your feedback. The team has made various edits to the overall structure of the manuscript based on your recommendations and have addressed some of your concerns.

I strongly recommend that authors amend the entire article to the form of a standard paper as Introduction, Material and Methods, Discussion and Conclusion.

The research team has restructured the manuscript to better adhere to PLOS ONE standards based on PLOS ONE guidelines and a review of other published manuscripts. The research team shortened the introduction. Instead of presenting the results of the prior work in the introduction, the research team moved it to the discussion section to more directly compare with our results.

1. Introduction with maximum 2 pages and containing the main objectives and justification;

While drafting the manuscript, the research team was not aware of this requirement and have adjusted the introduction to reduce its length to a more appropriate length. Thank you for bringing this to our attention.

2. I believe that the number of animals (n = 5) is quite small to have a reliable result with the main hypothesis of the study. If the authors want to keep this number, they will need to present the Test Power used in the methodology that indicated this minimum number of animals. It should be noted that sometimes the use of the minimum number of animals presented by the Power of Test is not ideal for the results to be faithful to the initial hypothesis of the study.

The research team acknowledges that the sample size used in this study is relatively small. A power test was conducted which determined between 15 and 30 animals would be required in order to achieve a statistical power of 0.8 to 0.95 across all tissues and tissue conditions. It is relatively common in animal-based exploratory studies to use a sample size lower than required for such statistical power. The research team has added verbiage to the conclusion section, in lines 419-421, of the manuscript stating this and refers to other cited work that makes use of similar sample sizes.

3. I strongly recommend that authors only perform tissue evaluation in vivo, ex vivo and post-freeze, as these are the most used methods in training; and in the following tissues: liver, spleen and lung.

The research team strove to be as comprehensive as possible with the resources available; the intermediate “*post mortem*" and “post-refrigeration” states have been included in the manuscript as they may provide useful insight and comparisons for future work.

The research team did also gather data from spleen and lung tissues, however there was insufficient analyzable data for our analysis methods. This is due to difficulties in handling the tissues, largely caused by limited tissue surface area for grasping. Only previously un-grasped areas were used, as mentioned in lines 300-301.
